# A further step towards early and systematic psychoeducation for caregivers with the BREF program

**DOI:** 10.1192/j.eurpsy.2023.698

**Published:** 2023-07-19

**Authors:** R. Rey

**Affiliations:** Centre Lyonnais des Aidants en Psychiatrie - CLAP, Centre Hospitalier Le Vinatier, Bron, France

## Abstract

**Introduction:**

Despite international and national guidelines advocating psychoeducation for caregivers (PEC) as one of the most effective treatments for patients living with a severe mental disorder (SMD), such programs remain scarce. Only 3% of the 4.5 million French caregivers in psychiatry have benefited from PEC and less than 10% know family associations (i.e., peer-led organizations supporting family members, caregivers, and loved ones of individuals living with a SMD). Worryingly, PEC is provided on average 10 years after the disease onset in France. Recognizing this major shortage in mental health organization, Rey et al. together with the Unafam family association created a short psychoeducational program called “BREF”. BREF means “brief” in French and can be provided early and systematically to caregivers of individuals with a SMD.

**Objectives:**

The aim of the present study was to assess the impact of the BREF program on depressive symptoms and burden of caregivers who benefited from the program.

**Methods:**

This is a retrospective, multicenter, open, uncontrolled study. 303 caregivers of persons living with a SMD benefited from the BREF program and were included in the present study. Depressive symptoms (assessed using the Center for Epidemiologic Studies Depression Scale, CES-D) and caregiver burden (assessed using the Zarit Burden Interview, ZBI) were measured before the BREF program, after the third session and during the 3‑month telephone callback. Quantitative data on caregivers’ satisfaction were collected at the 3‑month telephone callback.

**Results:**

The 303 caregivers included belonged to 216 families. Caregivers were mostly female (65.9%), they were mainly parents (66.8%) and spouses (17.3%). 20% of the included caregivers didn’t know the diagnosis of their relative and 69% had been caring for their relative for less than 5 years. As compared to baseline, we report a significant reduction in depressive symptoms and caregiver’s burden after the third session of the BREF program (p<0.001) and at the 3‑month telephone callback (p<0.05) (Fig. 1-2). The proportion of caregivers with a probable depression (CES-D≥20) was significantly lower after the third session of the BREF program (p=0.02) (Fig. 3). At 3 months, high levels of satisfaction were observed, with 98.4% of caregivers being satisfied or very satisfied with the BREF program. Caregivers deemed that the BREF program was very useful with a mean score of 9/10 (±1.5). 73% of the included caregivers attended the 3 sessions of the BREF program.

**Image:**

**Figure:**
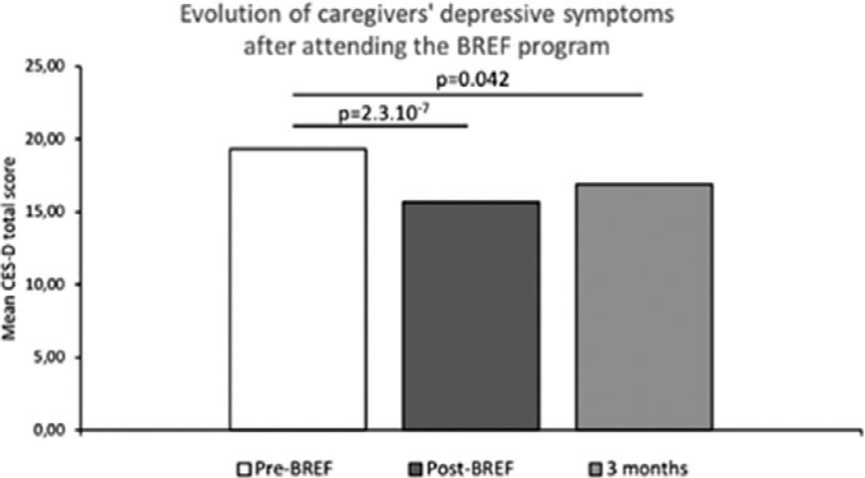

**Image 2:**

**Figure:**
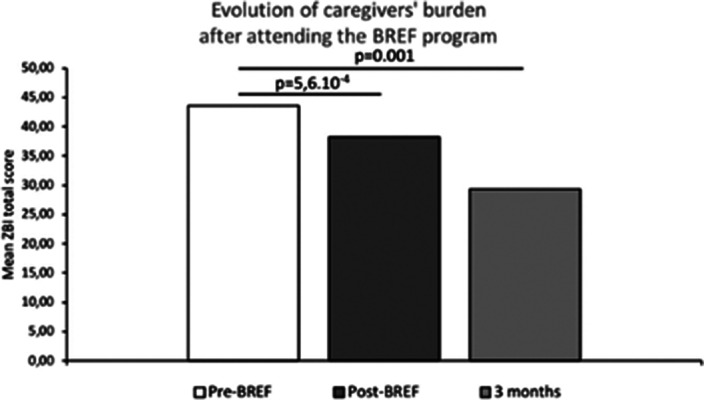

**Image 3:**

**Figure:**
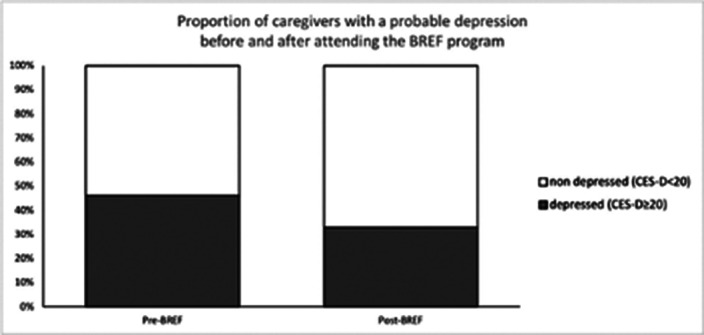

**Conclusions:**

The BREF program is associated with a therapeutic benefit for caregivers. In addition, BREF demonstrates a high level of caregiver satisfaction which is critical for a program intended to be provided early and systematically. The BREF program could reduce the French shortage in PEC provision. These results strongly support the national dissemination of the BREF program.

**Disclosure of Interest:**

None Declared

